# The Rights and Freedoms Gradient of Health: Evidence from a Cross-National Study

**DOI:** 10.3389/fpsyg.2012.00441

**Published:** 2012-11-07

**Authors:** Brent Bezo, Stefania Maggi, William L. Roberts

**Affiliations:** ^1^Department of Psychology, Carleton UniversityOttawa, ON, Canada; ^2^Department of Psychology, Institute of Interdisciplinary Studies, Carleton UniversityOttawa, ON, Canada; ^3^Department of Psychology, Thompson Rivers UniversityKamloops, BC, Canada

**Keywords:** physical health, mental health, health, rights, freedoms, gradient, social capital, socioeconomic status

## Abstract

This study examined the combined influences of national levels of socioeconomic status (SES), social capital, and rights and freedoms on population level physical and mental health outcomes. Indicators of mental health were suicide rates, alcohol consumption, and tobacco use. Indicators of physical health included life expectancy, infant mortality rates, and prevalence of HIV. Using pathway analysis on international data from a selected sample of European, North American, South American, and South Caucasus countries, similar models for mental health and physical health were developed. In the first model, the positive effects of SES and social capital on physical health were completely mediated via rights and freedoms. In the second model, the positive effect of SES on mental health was completely mediated, while the impact of social capital was partially mediated through rights and freedoms. We named the models, the “rights and freedoms gradient of health” in recognition of this latter construct’s crucial role in determining both physical and mental health.

## Introduction

Understanding how social and political forces determine population health outcomes across the life cycle is an overwhelming task. The diversity in the social and political systems that make up today’s global community makes this task even more daunting. However, despite this diversity, there is one pattern that has emerged in virtually all societies, and for a wide range of outcomes: the social gradient of health (Marmot et al., 1991; Deaton, 2002; Kosteniuk and Dickinson, 2003; Wilkinson and Picket, 2009). In the context of national trends, the gradient refers to an individual’s position in a socioeconomic hierarchy: individuals with less income are at greater risk for poorer health than individuals with greater income (Mathews and Gallo, 2011). By virtue of being a gradient, this principle applies to all income levels with not only the poorest being at greater risk of ill-health than the richest, but also with the well-off being at risk of poorer health than the richest. In the context of international trends, the gradient demonstrates that poorer countries have worse health outcomes than wealthier counties (Dasgupta and Weale, 1992).

Given the large body of evidence, this notion of a “social gradient of health” has become an accepted tenet in the field of social determinants. However, much debate ensues as to how the social gradient determines health outcomes (Wilkinson, 2000).

Considerable interest has been generated by the “*social inequality argument*” that draws attention to the steepness of the gradient line: the greater the income inequality, the steeper the social gradient of health. As such, the social inequality argument maintains that the wealthiest nations do not necessarily have the best health, but rather, that nations with the most egalitarian distribution of wealth have the best health outcomes (Marmot, 2002; Wilkinson and Picket, 2009).

In the context of child development, research suggests that early childhood is a particularly sensitive time for the determination of life-long health outcomes. Specifically, the prenatal, perinatal periods, and the early years have been shown to be the most sensitive to the effects of social inequality (Barker, 1997; Jefferis et al., 2002). Therefore, the available evidence pertaining to the social inequality argument reinforces the idea that economic factors, such as socioeconomic status (SES) and income distribution, account for a country’s life-long health outcomes.

In parallel, the “*civil society and social capital argument*” has also gained recognition in the field of health and its social determinants. This argument postulates that individuals are healthier where people collectively organize themselves in informal, non-governmental, and/or voluntary associations. According to the World Health Organization (WHO) Civil Society Initiative, “civil society is usually understood as the social arena that exists between the state and the individual or household. Civil society lacks the coercive or regulatory power of the state and the economic power of the market but provides the social power or influence of ordinary people” [World Health Organization (WHO), 2001, p. 3]. A closely related and much overlapping construct is that of social capital. Social capital is a multi-dimensional construct that has been described as the interaction between individuals in social associations or networks that generates cooperation and trust required for the realization of common goals (Putnam, 2000). Mounting empirical evidence has suggested that social capital is linked to better individual health outcomes at the community, regional, and other sub-national levels (Islam et al., 2006). For example, Maggi et al. (2011) found that preschool children had better mental health outcomes in communities where coalitions of early childhood educators were a stronger presence in the local community. However, cross-national research examining social capital and country-level health outcomes has either been relatively scarce or not necessarily shown positive results, especially in the context of developed countries (Kennelly et al., 2003). Yet, comparisons of developed and developing countries have shown a positive influence of social capital on physical health outcomes (Elgar, 2010).

Finally, supported by decades of cross-national research (Dasgupta, 1990; Frey and Al-Roumi, 1999; Franco et al., 2004; Altman and Castiglioni, 2009) the “*rights and liberties argument*” proposes that political rights and civil liberties are responsible for improvements in life expectancy and child survival. Political rights and civil liberties define democracy and freedom as the degree to which “the power of the elite is minimized and that of the non-elite is maximized” (Bollen, 1980, p. 372). Accordingly, political rights and civil liberties are organizational arrangements that reflect the extent to which power is distributed to non-elites (Bollen, 1986). Political rights allow citizens to choose who governs their nation and what laws and policies shall exist. Civil liberties include freedom of expression, association, speech, and media that are protected by the rule of law. As such, these liberties protect individuals’ expression of opinions without fear of reprisal from the state (Dasgupta, 1990). A population with greater civil liberties possesses a greater capacity to influence the decisions of the elite (Gearty, 2003). Directly connected to the rights and liberties argument is the ability of democracies to curb corruption (Drury et al., 2006), an important factor associated with infant and child mortality rates (Gupta et al., 2000).

**Figure 1:**
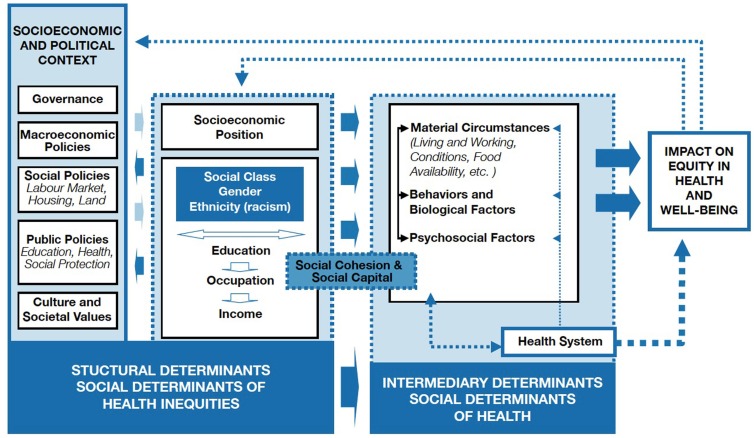
**WHO framework of social determinants of health [World Health Organization (WHO), 2010]**. Permission was granted to reproduce this diagram as it was originally published by the World Health Organization (WHO; 2010) on page 6 in A Conceptual Framework for Action on the Social Determinants of Health.

The three arguments of social inequality, civil society and social capital, and rights and liberties lend themselves to being integrated into one complementary view. It has been reasonably argued that in addition to influencing health outcomes directly, improvements in wealth distribution lead to gains in civil society and social capital, which in turn improve health outcomes of individuals and whole countries. This school of thought is evident in the social determinants of health framework put forward by the WHO Commission on the Social Determinants of Health, after much consideration of the extant interdisciplinary literature on the topic [World Health Organization (WHO), 2010]. As shown in Figure 1, socioeconomic and political factors are conceptualized as structural determinants, while the intermediary determinants are viewed as proximal influences (e.g., living and working conditions) and biological predispositions. Social cohesion and social capital are noted to act as influences between these structural and intermediary determinants.

In this model, the social inequality and rights and liberties arguments, although not explicitly embedded in its graphic representation, would be placed with the socioeconomic and political structural determinants. The civil society and social capital argument has been placed between the structural and intermediary determinants, acting somewhat as a mediator between the two. This framework, while acknowledging the bi-directionality of many of its influences, proposes a pathway leading to better health that commences from good governance and national wealth, leading to improvements in the social and socioeconomic conditions of individuals, who in turn are able to contribute to improvements in civil society and social capital. This WHO framework of the social determinants of health offers a logical and well-researched integration of the three explanatory arguments discussed above (i.e., social inequality, civil society and social capital, and rights and liberties) that may help to explain the determining forces behind the social gradient of health.

In this study, we applied the WHO framework to international data pertaining to 34 European, North and South American, and South Caucasus countries. We gathered indicators of physical and mental health outcomes pertaining to children (i.e., infant mortality), and youth and adults (i.e., suicide, tobacco and alcohol use, HIV/AIDS, and life expectancy). Our expectation was that, consistent with the WHO framework, good governance (rights and liberties) would promote gains in wealth and reductions in social inequality, which in turn would be associated with better health outcomes. We also expected civil society and social capital to moderate the effect of SES. In other words, we expected that wealthier countries with high social capital would have better health outcomes than better-off countries with low social capital.

## Materials and Methods

### Measures and sample

Data were obtained from online databases and publications of the WHO, World Bank, United Nations (UN), Freedom House, Transparency International, International Labour Organization (ILO), and the World Values Survey (WVS). Our full sample consisted of 34 countries from Europe, North and South America, and the South Caucasus that included (in alphabetical order): Albania, Argentina, Armenia, Brazil, Canada, Chile, Croatia, Czech Republic, Denmark, Estonia, Finland, France, Georgia, Germany, Greece, Hungary, Italy, Latvia, Lithuania, Mexico, Moldova, Netherlands, Norway, Poland, Portugal, Romania, Russia, Serbia, Slovenia, Spain, Sweden, Ukraine, United Kingdom, and United States of America (USA). As the WVS (that was used to study the role of social capital) did not have data for all of the nations in the full sample, we also conducted analysis on a subsample of 23 countries for which data on social capital was available. The 23 countries from Europe, North and South America, and the South Caucasus included (in alphabetical order): Argentina, Brazil, Canada, Chile, Finland, France, Georgia, Germany, Italy, Mexico, Moldova, Netherlands, Norway, Poland, Romania, Russia, Serbia, Slovenia, Spain, Sweden, Ukraine, United Kingdom, and United States of America.

### Socioeconomic status

Gross domestic product (GDP) per capita (US$), the Gini index, and unemployment were used to measure national levels of SES. GDP is defined as the total output of goods and services by a national economy. GDP per capita data, from 2008, were obtained from the World Bank Indicators Online (World Bank, 2012a) and are defined as the GDP divided by the national population.

Data for the Gini index were obtained from the World Bank’s World Development Indicators publication and were used to measure income inequality. A value of zero represents perfect income equality, whereas 100 represents absolute inequality (World Bank, 2011). The Gini data ranged from 1996 to 2009. However, only available data for the single, most recent year of any given nation were used. Approximately, 82.35% of Gini data were from the year 2000 or more recent.

Unemployment is considered one of the broadest economic indicators as reflected by the labor market (World Bank, 2011) with data obtained from the ILO’s Key Indicators of the Labor Market database [International Labour Organization (ILO), 2011]. Specifically, unemployment data represent the percent of the total labor force that is devoid of, but is available for and searching for, work. Data ranged between the years 2006 and 2010. Only unemployment data for the single, most recent year of any given nation were used.

### Social capital

Social capital indicators for membership in associations, trust, and political activism were obtained from the 2005–2008 wave of WVS (World Values Survey Association, 2008). The Survey’s association sources local teams to collect data in their respective countries, thereby monitoring international trends in values and beliefs. Every nation was surveyed once during the 2005–2008 wave. Our subsample of 23 nations with WVS data included 28,856 participants.

Measures assessing trust in institutions, that included the police, courts, government, the press, and political parties, were originally rated on four-point scales, with 1 and 2 indicating trust (“completely trust” or “trust somewhat”) and 3 and 4 indicating lack of trust (“do not trust very much” or “do not trust at all”). These scores were dichotomized so that 1 indicates complete or partial trust and 0 indicates complete or partial distrust. Thus, mean values for these variables are the proportion in the sample trusting a particular institution (press, police, courts, government, political parties). Political activity was assessed by self-reports of signing a petition, attending a demonstration, or participating in a boycott. Results were scored as 1 (=“have done”) or 0 (=“might do” or “would never do”). Respondents were asked if they were active or inactive members of nine different types of organizations. We grouped active and inactive responses together (=1), in contrast to those who indicated that they had never belonged to that type of organization (=0). We considered three different types of membership: churches or religious organizations; labor unions; and, all other organizations that included sports, arts or music, political parties, environmental, professional, charitable, or consumer organizations.

### Rights and freedoms

Measures of political rights, civil liberties, and corruption were used to evaluate rights and freedoms in the sample of nations. Indicators of democracy from the year 2008 were obtained from Freedom House based on their political rights and civil liberties indices (Freedom House, 2011a). The political rights index assesses the electoral process and free and fair elections, political pluralism, freedom to participate in the political process, and functioning and transparency of government. The civil liberties index assesses freedom of expression, belief, association, and organization. This latter index also assesses the rule of law, personal autonomy, and individual rights (Freedom House, 2011b). Both indices are rated on a 1–7 scale, with 1 being the highest and 7 being the lowest. However, for sake of interpretation, the original political rights and civil liberties scores were reflected where 1 represented low and 7 denoted high levels of political and civil liberties.

Measures of perceived corruption related to the public sector (Transparency International, 2012a) were obtained from Transparency International. The 2008 country scores were originally measured on a ten-point scale, where zero (0) denoted high and 10 indicated low levels of corruption (Transparency International, 2012b).

### Physical health

Measures of physical health included life expectancy, infant mortality rates, and prevalence of HIV in youth and adults. Life expectancies were obtained from the World Population Prospects database of the UN for the combined years of 2005–2010. Life expectancy at birth in years was calculated by the UN as “the average number of years of life expected by a hypothetical cohort of individuals who would be subject during all their lives to the mortality rates of a given period” [United Nations (UN), 2011]. Infant mortality rates, collected for the year 2010 from the World Bank’s online data base, are the number of infants dying prior to reaching 1 year of age per 1000 live births (World Bank, 2012b). HIV data were obtained from the UNAIDS Report on the Global AIDS Epidemic [United Nations (UN), 2010] and were defined as the estimated percentage of 15–49 year olds in the population living with HIV in 2009.

### Mental health

Mental health was assessed with measures of suicide rates, tobacco use, and alcohol consumption. International suicide rates per 100,000 were obtained from the WHO’s online mental health country charts [World Health Organization (WHO), 2012]. Suicide data ranged from 2003 to 2009. Only available data for the single, most recent year of any given country were used. Of the 34 nations, 82.35% of the suicide data were from the years 2007–2009.

Data for the percentage of national populations that engaged in current-daily tobacco-cigarette smoking were obtained from appendix 8 of the WHO’s Report on the Global Tobacco Epidemic [World Health Organization (WHO), 2011a]. This data did not represent youth tobacco use rates, although: 1 country reported for ages 11 and up; 1 country for ages 12 and up; 19 countries reported for ages 15 and higher; and, 4 countries reported tobacco use concerning individuals ages 16 and up. Tobacco data, for any given nation, were obtained for the single, most recent available year between 2000 and 2010; 76.47% of this data were from the years 2007–2010. Data for alcohol consumption was retrieved from the WHO’s Global Health Observatory Data Repository [World Health Organization (WHO), 2011b]. These 2005 data indicate the total recorded and unrecorded consumption per capita, in liters of pure alcohol, for ages 15 years and up.

### Procedure

For our path models, the latent variable partial least squares (LVPLS) approach developed by Lohmöller (1984) was used. According to Falk and Miller (1992) and Wold (1980), LVPLS is particularly appropriate when relations between theoretical constructs cannot be specified exactly, when empirical measures have some degree of unreliability, when there are many manifest and latent variables, and when sample sizes are moderate or small. Thus, this type of analysis is particularly appropriate given the conditions found in the present study.

Lohmöller’s (1984) approach does not generate standard errors for path coefficients (and hence tests of significance), on the grounds that this entails assumptions about the multivariate distributions that are difficult to test and unlikely to be true (Wold, 1980; Falk and Miller, 1992). Thus, we required that path coefficients and factor loadings be of at least moderate size. For example, Falk and Miller (1992) recommend factor loadings of at least 0.55, indicating that the manifest variable shares 30% of its variance with its latent variable. This criterion was met in our models with only two exceptions, as is revisited in the results. For path coefficients, we set a minimum size of 0.30. In contrast to path coefficients and factor loadings, we could and did apply tests of significance to the *R*^2^ values generated by our models. All were significant, *p*s < 0.001.

Because path analysis requires complete data, missing values were estimated within domains if only one measure was missing. For example, if a country was missing one of three measures of mental health (such as tobacco use), that missing value was estimated by maximum likelihood procedures (BMDP AM, Dixon, 1992) using the other two mental health measures (alcohol consumption and suicide rate). Although this strategy slightly inflates coherence within domains, it does not affect relations between domains. For mental health, two countries were missing data for tobacco use and two others for alcohol consumption. For physical health, two countries were missing data for HIV infection rates. All countries had complete data for SES, and rights and freedoms. For the subsample, all 23 countries had complete social capital data.

## Results

This section begins with descriptions of the sample countries in terms of our variables of interest – SES, social capital, rights and freedoms, and physical and mental health. Next, the relations within and between these domains are examined, first by considering correlations between each of the individual measures, and second by aggregating these measures on latent variables using path analysis. Separate path models were developed for physical and mental health; and for each health outcome, relations were examined in the full sample of 34 countries and the subsample of 23 countries for which social capital data were available. For each of these four models, we examined the issue of whether, as we expected, SES directly predicted health outcomes, mediating relations between health and rights and freedoms (and social capital, when it could be examined). As seen below, although relations between SES and health were substantial, relations between rights and freedom and health were even stronger, and our analyses indicated that rights and freedoms mediated the effects of SES rather than vice versa. Similarly, in our subsample of 23 countries, we examined the issue of whether social capital directly predicted health, or whether the effects of social capital were mediated by rights and freedoms. As seen below, the answer to this question differed according to the health outcomes we considered, physical or mental.

### Descriptive findings

#### Socioeconomic status

As shown in Table 1, for these 34 nations, the mean GDP per capita was USD 26,048.01 (SD = 21,688.87). For the subsample of 23 nations, the mean was USD 28,814.15 (SD = 23,760.92). For both the full and subsamples, GDP per capita ranged from USD 1,696.0 (Moldova) to USD 95,189.9 (Norway). For the full sample, the mean Gini index was 34.13 (SD = 7.22); for the reduced sample, the mean was 34.68 (SD = 8.25). For both samples, Brazil had the highest Gini index of 53.9, while Chile had the lowest of 22.6.

**Table 1:** **Descriptive statistics *N*s = 34 and 23**.

Variable	Mean	SD	Minimum	Maximum
***N* = 34**				
SES				
GDP (per capita, USD)	26,048.01	21,688.87	1,696.0	95,189.9
GINI	34.13	7.22	22.6	53.9
Rights and freedoms				
Corruption	5.56	2.18	2.1	9.3
Political rights	6.2	1.4	2	7
Civil liberties	6.3	1.1	3	7
Mental health				
Suicide (per 100,000)	13.39	7.71	1.9	34.1
Alcohol consumption	17.92	5.21	8.68	27.91
Tobacco (% daily use)	23.59	6.45	7.6	40.2
Physical health				
Life expectancy (years)	76.25	3.98	67.54	81.37
Infant mort (per 1,000)	7.31	5.27	2.3	20.0
HIV (% prevalence)	0.31	0.31	<0.1	1.2
***N* = 23**				
SES				
GDP (per capita, USD)	28,814.15	23,760.92	1,696.0	95,189.9
GINI	34.68	8.25	22.6	53.9
Social capital				
% Trust police	56.0	21.5	19.9	91.5
% Trust courts	46.1	18.6	19.5	86.0
% Politically active	44.7	21.5	9.3	80.4
% Church members	35.9	21.5	7.5	78.5
% Union members	19.9	14.1	3.2	58.5
% Members of other org.	45.0	21.4	2.7	83.5
Rights and freedoms				
Corruption	5.73	2.39	2.1	9.3
Political rights	6.2	1.3	2	7
Civil liberties	6.2	1.2	3	7
Mental health				
Suicide (per 100,000)	12.73	6.51	4.2	30.1
Alcohol consumption	17.26	5.49	8.68	27.91
Tobacco (% daily use)	22.18	6.31	7.6	33.8
Physical health				
Life expectancy (years)	76.60	4.47	67.54	81.37
Infant mort (per 1,000)	7.58	5.37	2.3	20.0
HIV (% prevalence)	0.32	0.28	<0.1	1.1

*For the analyses, we substituted a point-estimate of 0.05 for the approximate value of <0.1 given in our sources. Lacking more precise information, this seemed a reasonable estimate because 0.05 is halfway between 0 and 0.1, and so unknown values <0.1 might be expected to center on it*.

#### Social capital

As shown in Table 1, police were the most trusted social institution (over half of WVS respondents), followed by the courts. Nearly half of respondents reported engaging in at least one political activity (signing a petition, attending a demonstration, or engaging in a boycott). Most people (60%) belonged or had belonged to organizations, but over a third of these (22.5% of the sample) reported that they were actively involved with only one group. Fewer than 10% of the sample were active in two groups, and fewer than 8% were actively involved with three or more groups. Considering active and inactive memberships together, over a third of the sample reported membership in religious organizations and nearly a fifth in labor unions (see Table 1). In the category of “other organizations,” membership was most common in sports groups (24.8%), art/music/education groups (16.8%), and charities (16.5%), followed by professional organizations (12.8%) and political parties (11.1%).

#### Rights and freedoms

Of the 34 nation full sample, the average political rights score was 6.2 (SD = 1.4), while the average civil liberties score was 6.3 (SD = 1.1). In the full sample, several countries including Canada, Chile, Czech Republic, Denmark, Estonia, Finland, France, Germany, Hungary, Lithuania, Netherlands, Norway, Poland, Portugal, Slovenia, Spain, Sweden, United Kingdom, and USA were ranked as 7 for the highest level for both political rights and civil liberties. Russia was ranked the lowest for both political rights (2) and civil liberties (3). Of the 23 nation subsample, the average political rights score was 6.2 (SD = 1.3); the average civil liberties score was 6.2 (SD = 1.2). Of the subsample, Canada, Chile, Finland, France, Germany, Netherlands, Norway, Poland, Slovenia, Spain, Sweden, United Kingdom, and USA were tied for the highest level (7) for both political rights and civil liberties. In the reduced sample, Russia was ranked the lowest for political rights and civil liberties with scores of 2 and 3, respectively.

The average corruption score was 5.56 (SD = 2.18) for the 34 nation full sample. Specifically, Denmark and Sweden were tied for the lowest level of perceived corruption with a score of 9.3. Russia had the highest perceived level of corruption with a score of 2.1. Of the 23 nation subsample, the average corruption score was 5.73 (SD = 2.39). Of the subsample, Sweden was rated as the least corrupt with a score of 9.3, while Russia was rated as the most corrupt with a score of 2.1.

#### Physical health

The average life expectancies were 76.25 (SD = 3.98) and 76.60 (SD = 4.47) years in the full and subsamples, respectively. Of both samples, Italy had the longest life expectancy with 81.37, while Ukraine had the lowest with 67.54 years. The average infant mortality rates were 7.31 (SD = 5.27) and 7.58 (SD = 5.37) per 1000 births for the full and subsamples, respectively. Of both samples, Slovenia had the lowest infant mortality rate of 2.3 per 1000 births, while Georgia had the highest of 20.0. Of the 34 nation full sample, the average prevalence of HIV was 0.31% (SD = 0.31). Several countries, including Croatia, Czech Republic, Hungary, and Slovenia were tied for the lowest prevalence with less than 0.1% of the population living with HIV. The nation with the highest prevalence was Estonia with 1.2% of the population living with HIV. Of the 23 nation subsample, the average prevalence of HIV was 0.32% (SD = 0.28). In this latter sample, Slovenia had the lowest HIV prevalence (<0.1%), while Ukraine had the highest (1.1%).

#### Mental health

Of the 34 nations that comprised the full sample, the average suicide rate was 13.39 per 100,000 (SD = 7.71). Armenia had the lowest suicide rate with 1.9, while Lithuania had the highest occurrence with 34.1 per 100,000. The average suicide rate for the 23 nation subsample was 12.73 (SD = 6.51) per 100,000. Of the subsample, Mexico had the lowest suicide rate with 4.2, while Russia had the highest with 30.1 per 100,000. The average percent of tobacco-cigarette smokers in the full sample was 23.59% (SD = 6.45). Of the full sample, Mexico had the lowest (7.6%) percentage of tobacco users; Albania had the highest (40.2%). The average percent of tobacco-cigarette smokers in the subsample was 22.18% (SD = 6.31). Of the subsample, Russia had the highest percentage of adult tobacco users (33.8%), while Mexico had the lowest (7.6%). The average total alcohol recorded and unrecorded consumption per capita was 17.92 liters (SD = 5.21) and 17.26 liters (SD = 5.49) for the full and subsamples, respectively. For both samples, Ukraine had the highest level of consumption in liters (27.91); Norway had the lowest (8.68).

### Relations between SES, social capital, rights and freedoms, and health: Correlations

Our measures of national level SES showed only moderate convergence. As shown in Table 2, greater GDP was significantly and moderately related to lower levels of economic inequality (Gini) and lower rates of unemployment, as expected. In contrast, unemployment and economic inequality were unrelated.

**Table 2:** **Correlations between variables**.

Variables	1	2	3	4	5	6	7	8	9	10	11	12	13	14	15	16	17
**SES**																	
GDP	1.00																
GINI	−0.40*	1.00															
Unemploy.	−0.41*	0.03	1.00														
**SOCIAL CAPITAL**																	
Trust police	0.85***	−0.45*	−0.09	1.00													
Trust courts	0.86***	−0.30	−0.21	0.87***	1.00												
Pol. active	0.79***	−0.12	−0.16	0.79***	0.78***	1.00											
Church	0.25	0.24	−0.33	0.31	0.51*	0.45*	1.00										
Union	0.61**	−0.42*	−0.37+	0.52*	0.75***	0.51*	0.50*	1.00									
Other org	0.75***	−0.19	−0.48*	0.64***	0.72***	0.84***	0.63**	0.67***	1.00								
**RIGHTS AND FREEDOMS**																	
Corruption	0.83***	−0.49**	−0.31+	0.85***	0.73***	0.72***	0.37+	0.54**	0.73***	1.00							
Pol. rights	0.55***	−0.25	−0.37*	0.64***	0.48*	0.59**	0.38+	0.25	0.59**	0.69***	1.00						
Civil lib.	0.54***	−0.41*	−0.20	0.62**	0.47*	0.57**	0.29	0.30	0.54**	0.72***	0.92***	1.00					
**MENTAL HEALTH**																	
Suicide	−0.10	−0.19	−0.01	−0.20	−0.09	−0.27	−0.29	0.21	−0.16	−0.06	−0.02	0.12	1.00				
Alcohol	−0.57***	0.29+	0.11	−0.66***	−0.46*	−0.66***	−0.23	−0.34	−0.54**	−0.55***	−0.36*	−0.35*	0.31+	1.00			
Tobacco	−0.44**	−0.24	0.34*	−0.32	−0.48*	−0.58**	−0.78***	−0.40+	−0.68***	−0.44**	−0.41*	−0.32+	0.28	0.15	1.00		
**PHYSICAL HEALTH**																	
Life expect.	0.72***	−0.33+	−0.24	0.78***	0.58**	0.70***	0.26	0.28	0.64***	0.76***	0.68***	0.59***	−0.45**	−0.60***	−0.42*	1.00	
Infant mort.	−0.66***	0.52**	0.26	−0.67***	−0.57**	−0.54**	−0.01	−0.42*	−0.55**	−0.69***	−0.72***	−0.75***	−0.31+	0.21	0.21	−0.62***	1.00
% HIV	−0.22	0.28	0.05	−0.35+	−0.26	−0.25	−0.15	−0.20	−0.23	−0.22	−0.21	−0.13	0.25	0.40*	0.07	−0.43*	0.11

*N* = 23 for correlations with social capital; for all other variables, *N* = 34. +*p* < 0.10; *p < 0.05; **p < 0.01; ***p < 0.001. All tests are two-tailed. The binomial probability of observing 86 or more significant correlations in a set of 153 with alpha = 0.05 is <0.0001. This table uses 2008 data for GDP and rights and freedoms. Corruption is reverse-scored so that high scores = low levels of corruption. Suicide rate = rate per 100,000. Alcohol = alcohol consumption. Tobacco = % prevalence of daily tobacco use. Life expect = life expectancy in years. Infant mort = infant mortality per 1,000 live births. % HIV = % prevalence in population.

With the exception of church membership, our measures of social capital were all significantly and strongly related to one another and to GDP (all correlations >0.50; Table 2). In contrast, correlations between social capital and economic inequality and unemployment were less consistent and only moderately strong. Specifically, only five of ten comparisons were greater than 0.30, and all were less than 0.50.

The same pattern was shown by our three measures of rights and freedoms. Corruption, political rights, and civil liberties were strongly and significantly correlated with one another and with GDP, whereas correlations with economic inequality and unemployment were moderate and sometimes failed to reach statistical significance. Specifically, only four of six comparisons were greater than 0.30, and all were less than 0.50. Given their similar means and standard deviations (Table 1) and the very high correlation between civil liberties and political rights (see Table 2; in the subsample, *r* = 0.94), these two variables were averaged before entry into the path models.

As expected, many of the social capital variables were strongly and consistently related with our measures of rights and freedoms.

Our measures of mental health showed only weak convergence. Although suicide rates correlated about 0.30 with both alcohol consumption and tobacco use (and about 0.40 in the set of 23 countries for which we had social capital data), alcohol consumption and daily tobacco use showed little relation with one another in either the full or subsample. However, alcohol consumption and daily tobacco use were very similarly related to SES, rights and freedoms, and social capital. The median absolute correlations with these variables were 0.41 and 0.42, respectively, for alcohol and tobacco use. In contrast, suicide rate correlated with SES, rights and freedoms, and social capital at only chance levels.

A similar pattern within and across variables was seen for physical health. As expected, life expectancy showed good convergence with infant mortality and HIV rates. However, infant mortality and HIV were only weakly related to one another in the full and subsamples (Table 2; in the subsample, *r* = 0.28). And whereas life expectancy and infant mortality were both frequently and strongly related to SES, rights and freedoms, and social capital (8 of 12 correlations were greater than 0.50 for both variables), relations between these variables and HIV rates were at chance levels. Thus, within each set of three outcomes for physical and mental health, correlations indicated somewhat different patterns of association with our set of predictors.

### Relations between SES, social capital, rights and freedoms, and health: Path models

#### Mediated and direct relations: SES and rights and freedoms

**Figure 2:**
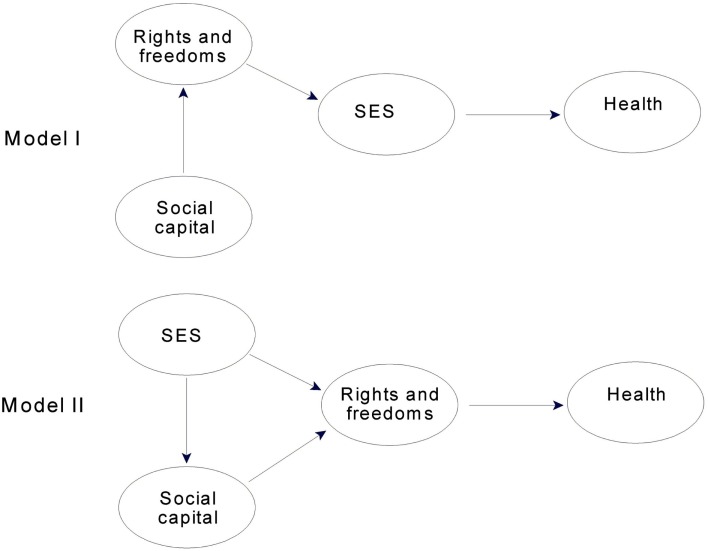
**Contrasting conceptual path models for evaluating direct and mediated effects for SES, and rights and freedoms for the 23 countries that had social capital data**. Models for the full sample omitted the latent construct for social capital.

We began by considering models that were consistent with our theoretical expectations, namely, that SES was a direct predictor of health and mediated the effects of rights and freedoms. First in the subsample and then in the full sample, we asked whether SES or rights and freedoms had a stronger direct effect on health, and then whether any evidence existed for the direct effect of the weaker variable when the stronger variable was already a direct predictor. In these contrasting models (see Figure 2), social capital when present, always predicted rights and freedoms.

For the 23 countries for which we had data on social capital, rights and freedoms was a stronger predictor of health than SES, particularly for physical health. Consistent with its importance in other studies, SES predicted 64% of the variance in physical health in a path model in which it directly predicted physical health (Model I, Figure 2). But rights and freedoms proved to be even stronger, predicting 79% of the variance in physical health when it was the direct predictor (Model II, Figure 2). For mental health, SES directly predicted 41% of the variance (Model I) and rights and freedoms predicted 49% (Model II). Thus, these initial findings in the subsample were consistent with models in which rights and freedoms directly influenced both physical and mental health and in each case mediated the effects of SES.

We then examined a third model, one in which SES and rights and freedoms jointly predicted health. Specifically, in Model II, Figure 2, a direct path was added from SES to health. In this model, SES increased the explained variance in physical health by less than 2% in comparison to rights and freedoms alone, and path coefficients of 0.72 for rights and freedoms and 0.21 for SES indicated a minimal role for SES as a joint predictor. The joint model for mental health could not be interpreted because of suppressor effects for the SES-mental health path (as indicated by different signs for the path coefficient and the correlation between the latent variables). Thus, no joint predictor model indicated an important direct effect for SES once rights and freedoms was a direct predictor of health.

These analyses were then repeated using all 34 countries in our sample. The pattern just described for the subsample was found again: SES was a strong predictor of physical and mental health, but rights and freedoms was even stronger, particularly for physical health. For the model in which only SES directly predicted physical health (Model I, Figure 2, social capital omitted), SES accounted for just under 60% of the variance in physical health. In contrast, for the model in which only rights and freedoms directly predicted physical health (Model II, Figure 2, social capital omitted), rights and freedoms predicted 71% of the variance. For mental health, the respective values were 34% for SES in Model I and 39% for rights and freedoms in Model II. Therefore, findings in the full sample, like those in the subsample, indicated that rights and freedoms directly influenced health and mediated the effects of SES, rather than vice versa.

As the final step in assessing mediated and direct relations for SES and rights and freedoms, we examined in our full sample the model in which SES and rights and freedoms were joint predictors of health. In this model, SES increased the explained variance in physical health by 3.8% in comparison to rights and freedoms alone, and path coefficients of 0.59 for rights and freedoms and 0.32 for SES indicated a substantially stronger relation for rights and freedoms than for SES. In the joint model for mental health, SES increased explained variance by only 1% in comparison to rights and freedoms alone, and path coefficients of 0.51 for rights and freedoms and only 0.15 for SES indicated a very minimal role for SES. Thus, joint predictor models using the full sample were inconsistent in their implications. The model for mental health indicated no important direct effect for SES independent of rights and freedoms. In contrast, the model for physical health indicated a modest role for SES as a joint predictor, in that its path coefficient was greater than 0.30. But since this relation was not large and did not replicate in the subsample (the path coefficient for SES was only 0.21 in the joint model, as noted earlier), we concluded that the evidence for a joint role for SES in predicting physical health was insufficient.

In sum, although SES was strongly linked to physical and mental health outcomes in our sample, consistent with results from other samples, relations between rights and freedoms and health were even stronger and entirely (in the case of mental health) or almost entirely (in the case of physical health) mediated the effects of SES.

#### Mediated and direct relations: social capital and rights and freedoms

**Figure 3:**
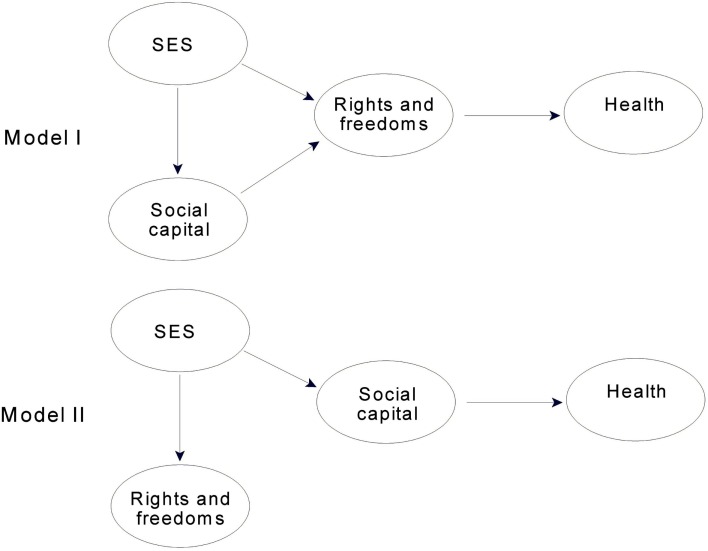
**Contrasting conceptual path models for evaluating direct and mediated effects for social capital, and rights and freedoms for the 23 countries with available social capital data**.

We approached the issue of direct and mediated relations for rights and freedoms and social capital using the same strategy as we did for SES and rights and freedoms, contrasting the path models shown in Figure 3. These contrasts, of course, could only be carried out in the subsample.

In the 23 countries for which we had social capital data, rights and freedoms clearly mediated relations between social capital and physical health. In Model I in Figure 3, rights and freedoms predicted 79% of the variance in physical health. The comparable value for social capital (Model II, Figure 3) was 61%. A third path model in which social capital was a direct, joint predictor of physical health (Model I with an added direct path from social capital to health) accounted for only 0.3% more variance in physical health than did rights and freedoms alone; and the small path coefficient for social capital (0.09) indicated that its role as a joint predictor was unimportant. Therefore, in the final model described below, the relation between social capital and physical health is mediated by rights and freedoms.

A different picture emerged when we considered relations between social capital, rights and freedoms, and mental health. When rights and freedoms directly predicted mental health (Model I, Figure 3), it accounted for 49% of the variance. When social capital directly predicted mental health (Model II, Figure 3), it was slightly stronger, accounting for 52% of the variance. A third path model in which rights and freedoms and social capital were joint predictors of mental health accounted for 54% of the variance. Although the increase over Model II was marginal (2%), the path coefficients were essentially equal (0.40 for social capital and 0.37 for rights and freedoms), indicating that both were important as joint predictors. Therefore, in the final model, described below, social capital and rights and freedoms both predict mental health. Because we had assessed mediation for SES using models in which the effects of social capital on mental health were mediated by rights and freedoms, we revisited this issue by assessing whether SES was an important direct predictor of mental health when social capital and rights and freedoms were joint predictors. The same pattern again emerged in this analysis: the direct path from SES to mental health had a suppressor effect, rendering the path model uninterpretable. Thus, there was no evidence to support a direct path between SES and mental health that was independent of rights and freedoms.

#### The final path models

This section describes the final models that emerged from our consideration of direct and mediated relations above, beginning with models for mental health. First, various aspects of fit between the models and the data are considered. Second, detailed descriptions of various aspects of the models, themselves, are presented.

**Figure 4:**
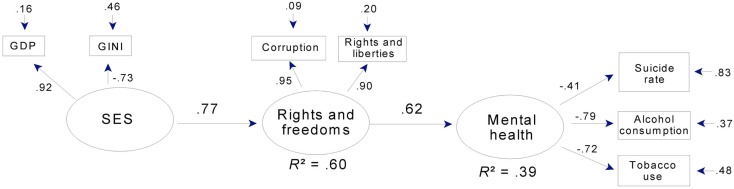
**Mental health, SES, and rights and freedoms: a path model for 34 countries**. Note that corruption is reverse-scored, so that high scores = low levels of corruption. Rights and liberties = mean ratings for political rights and civil liberties (see text). Gini: high scores = economic inequality. GDP = Gross domestic product per capita.

Mental health was strongly predicted in the full sample (Figure 4), where rights and freedoms predicted over a third of the variance in a latent variable aggregating tobacco use and alcohol consumption and (to a lesser extent) suicide rate.

**Figure 5:**
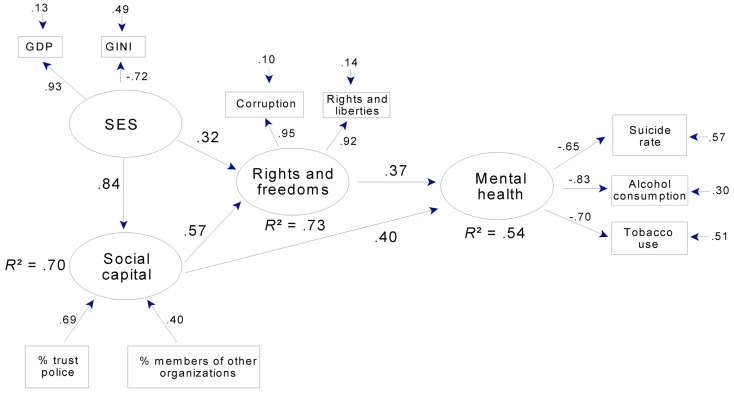
**Mental health, social capital, SES, and rights and freedoms: a path model for 23 countries**. Note that corruption is reverse-scored, so that high scores = low levels of corruption. Rights and liberties = mean ratings for political rights and civil liberties (see text). Gini: high scores = economic inequality. GDP = Gross domestic product per capita. % trust police = % of WVS sample expressing trust in police. % members of other organizations = % of WVS sample who were active or inactive members in non-religious, non-labor organizations.

In the subsample, rights and freedoms together with social capital predicted over half the variance in mental health (Figure 5). In both models, well over half the variance in rights and freedoms was predicted by SES or by SES together with social capital.

In addition to strong relations between latent variables, the final models for mental health adequately summarized our manifest variables. Mean communality was 0.63 in the full sample and was even higher in the subsample (0.71). The exception to this pattern of uniformly adequate communalities was the relation between latent mental health and suicide rate in the full sample (factor loading = −0.41; Figure 4). Suicide rate was retained in this model, however, in order to facilitate comparison between the full and subsamples (in Figure 5 the factor loading is −0.65) and because a loading of −0.41 seemed large enough to be of theoretical interest, since both the loading and the error term indicate that latent mental health in this model predicted 17% of the variance in suicide rate.

In contrast to levels of prediction and communality, levels of overall fit were moderate for both mental health models. The root mean square of the variance between the residuals of the manifest variables and the residuals of the latent variables [RMS COV (*e*,*u*); Falk and Miller, 1992], which reflects the amount of variance *not* accounted for by the model, was 0.12 for the full sample model and only slightly better in the subsample (0.11). Reflecting the fact that the models in Figures 4 and 5 specify most of the available paths, these RMS COV (*e*,*u*) values were close to those for the corresponding full models (that is, models that have all possible paths specified). They also represent a substantial improvement over the corresponding null models (that is, models in which no paths are specified). For these null models, RMS COV (*e*,*u*) was 0.23 and 0.24, respectively, for the full and subsamples. Thus, while the overall levels of fit were moderate at 0.11 and 0.12, this represents a substantial improvement over the respective null alternatives.

**Figure 6:**
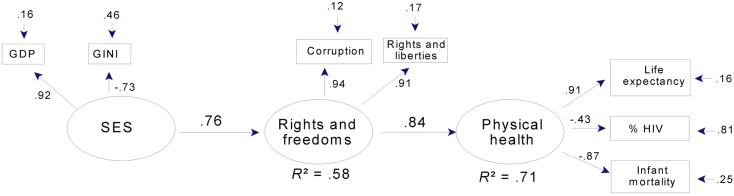
**Physical health, SES, and rights and freedoms: a path model for 34 countries**. Note that corruption is reverse-scored, so that high scores = low levels of corruption. Rights and liberties = mean ratings for political rights and civil liberties (see text). Gini: high scores = economic inequality. GDP = Gross domestic product per capita.

**Figure 7:**
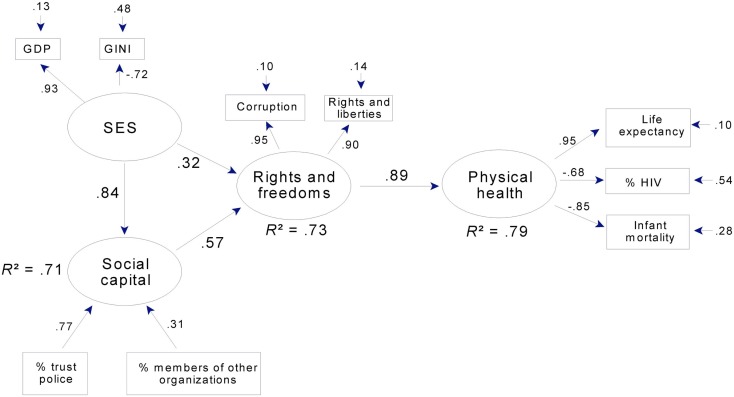
**Physical health, social capital, SES, and rights and freedoms: a path model for 23 countries**. Note that corruption is reverse-scored, so that high scores = low levels of corruption. Rights and liberties = mean ratings for political rights and civil liberties (see text). Gini: high scores = economic inequality. GDP = Gross domestic product per capita. % trust police = % of WVS sample expressing trust in police. % members of other organizations = % of WVS sample who were active or inactive members of non-religious, non-labor organizations.

In both mental health models, latent SES accounted for over 80% of the variance in GDP and more than half the variance in Gini scores. This latter pattern was also seen in the models for physical health (Figures 6 and 7). Hence, across all models, higher SES indicated higher per capita GDP and less economic inequality.

Latent rights and freedoms were strongly related to corruption and political rights and civil liberties, as indicated by factor loadings greater than 0.90 in all four models. Corruption was reverse-scored, so that higher values and positive factor loadings indicate lower levels of corruption. Thus, in all path models, high scores on rights and freedoms indicate low levels of corruption and high levels of political rights and civil liberties.

For both mental (Figure 5) and physical health (Figure 7), latent social capital was indexed primarily by the proportion of the WVS sample indicating trust in the police force, and to a lesser extent, by the proportion belonging to non-church, non-labor organizations. Their relative importance is indicated by their respective loadings or weights which are equivalent to standardized regression coefficients in a multiple regression analysis and, therefore, index or predict the outcome without presumptions about their causal origins or relations. In contrast, loadings for the “outer directed” latent variables (SES, rights and freedoms, health) are factor loadings – the correlations between the manifest variables and the latent variables they define. Thus, each latent variable summarizes the variance shared across the set of manifest variables from which it is derived, and this variance is shared presumably because the manifest variables share underlying causes (Shipley, 2000). For example, those factors (whatever they are) that affect physical health in a given society have an impact on both life expectancy and infant mortality, which consequently are correlated. In contrast, the manifest variables associated with an “inner directed” construct like social capital are not presumed to share variance because they share underlying causes. Therefore, other measures of social capital (e.g., trust in government, press, political parties) were not included in the models, as they were not related to rights and freedoms independently of trust in the police and rate of membership in other organizations due to their small weights (less than 0.30) in the preliminary path models.

In the models for mental (Figure 5) and physical health (Figure 7), social capital and SES were strongly related in that they shared over two-thirds of their variance. Although there are no clear theoretical grounds for specifying the causal influences between these two constructs, we chose to include a provisional path from SES to social capital, as discussed later. Omitting a path altogether would imply that there are *no* causal relations between social capital and SES, which is not plausible; and empirically, omitting a path between these two constructs results in a substantial increase in the model’s lack of fit, increasing RMS COV (*e*,*u*) for mental health, for example, to 0.14 from 0.11. Thus, the existence of a path is indicated, even if its direction is uncertain.

Overall, the path models in Figures 4 and 5 indicate strong relations between rights and freedoms and mental health, especially as indexed by lower alcohol consumption and reduced daily tobacco use. Figure 5 indicates that social capital is also a strong predictor of mental health, both directly and indirectly through rights and freedoms. SES is an important component of both models, strongly related to social capital, directly and indirectly related to rights and freedoms, and through rights and freedoms and social capital to mental health.

Physical health, like mental health, was strongly predicted in the full sample (Figure 6), where rights and freedoms predicted over two-thirds of the variance in a latent variable aggregating life expectancy, infant mortality, and (to a lesser extent) HIV prevalence. In the subsample, rights and freedoms together with social capital predicted over three-quarters of the variance in physical health (Figure 7). In both models, as in the mental health models, well over half the variance in rights and freedoms was predicted by SES or by SES together with social capital; and SES strongly predicted social capital.

In addition to strong relations between latent variables, the final models for physical health adequately summarized the manifest variables. Mean communality was 0.69 in the full sample model and was even higher in the subsample model (0.76). The exception to this pattern of uniformly adequate communalities was the relation between latent physical health and HIV prevalence in the full sample (factor loading = −0.43; Figure 6). Prevalence of HIV was retained in this model for the same reasons that suicide rate was retained in the model for mental health, that is, to facilitate comparison between the full and subsample models (in Figure 7 the factor loading for HIV prevalence is −0.68) and because a loading of −0.43 was large enough to be of theoretical interest. Both the loading and the error term indicate that latent physical health predicted 19% of the variance in HIV prevalence.

As with levels of prediction and average communality, levels of overall fit were adequate for both physical health models. RMS COV (*e*,*u*) was 0.07 for both the full and subsample models. Reflecting the fact that the models in Figures 6 and 7 specify most of the available paths, these RMS COV (*e*,*u*) values were close to those for the corresponding full models. They also represent a substantial improvement over the corresponding null models for the full and subsamples, which had RMS COV (*e*,*u*) values of 0.28 and 0.27, respectively.

In summary, the path models in Figures 6 and 7 indicate very strong relations between rights and freedoms and physical health, especially as indexed by life expectancy and low infant mortality. Figure 7 indicates that social capital had an important indirect relation to physical health through rights and freedoms. Finally, as in the models for mental health, SES was related to both social capital and rights and freedoms, and thus had, like social capital, important indirect links to physical health.

### The rights and freedoms gradient of health

**Figure 8:**
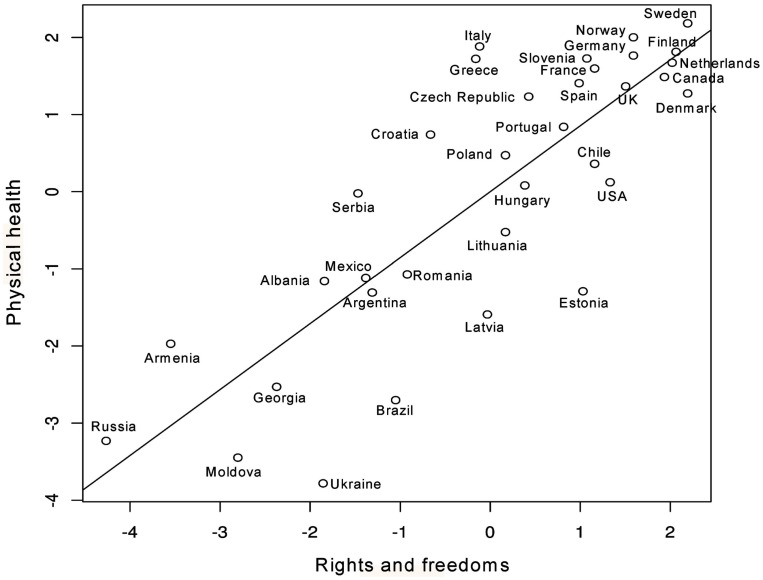
**Scatterplot of rights and freedoms versus physical health**.

**Figure 9:**
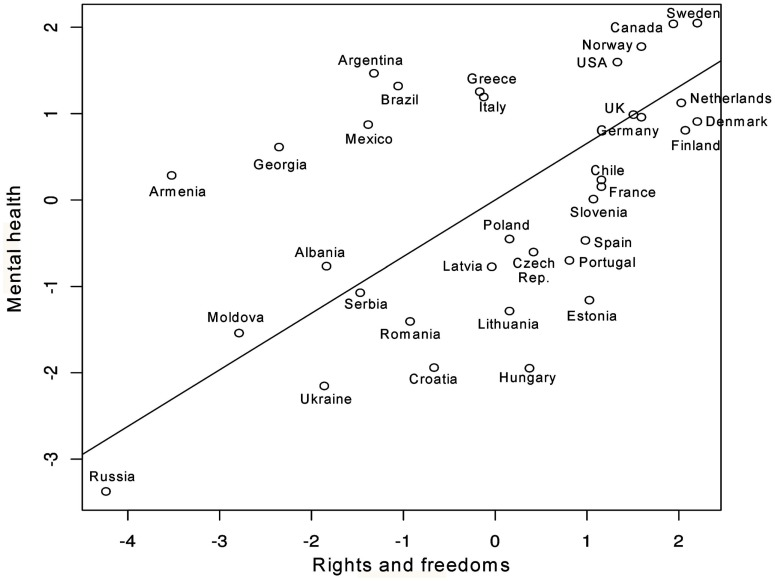
**Scatterplot of rights and freedoms versus mental health**.

Our results suggest that rights and freedoms have a prominent role in the determination of health outcomes, accounting for most of the association between SES and health, both physical and mental. In addition, rights and freedoms accounted for most of the association between social capital and physical health. Also, the positive effect of social capital on mental health was partially accounted for by rights and freedoms. These important and novel observations provide evidence for a rights and freedoms gradient in health, as represented in Figures 8 and 9. What Figures 8 and 9 indicate, for the most part, is that in countries where more rights and freedoms exist, children, youth, and adults have better physical and mental health outcomes. However, in a few exceptions, a small number of countries deviate from the gradient line, indicating either better or worse health outcomes than expected. To determine which country fell off the diagonal line we calculated *z* scores and looked for countries whose values significantly deviated from 0 (any value of more than 1.96 was considered significant at 0.05; and values of 1.645 significant at 0.10). This analysis led us to the identification of: (1) resilient countries such as Italy and Greece with better physical health outcomes and Argentina with better mental health outcomes than otherwise expected based on their scores in rights and freedoms; and (2) vulnerable countries, such as Ukraine, Estonia, and Brazil with worse off outcomes in physical health, and Hungary with worse off outcomes in mental health than otherwise expected based on their scores in rights and freedoms. The implications of this emerging evidence of a rights and freedoms gradient of health, and its interpretation in the context of cross-national research are discussed below.

## Discussion

In conducting this study, we set out to apply the WHO framework of social determinants (Figure 1) of health to a cross-national sample of 34 countries from Europe, North and South America, and the South Caucasus for which we had mental and physical health outcome data. The full sample of 34 countries, for which social capital data were unavailable, indicated significant positive effects of rights and freedoms on mental and physical health. We replicated the same models on a subsample of 23 countries for which we also had social capital data. Both samples contained developed countries and countries in transition. We anticipated that our results would be congruent with the WHO framework of social determinants (Figure 1). The WHO framework (Figure 1) associates increased rights and freedoms with increased wealth and social equality, which in turn promotes better health. Based on the WHO framework (Figure 1), we also anticipated that social capital and civil society would moderate the effect of SES. As earlier noted, this WHO framework (Figure 1) encapsulates the “*social inequality*,” “*civil society and social capital*,” and “*rights and liberties*” arguments. Therefore, similar to previous studies, we found that SES and social capital played important roles in the determination of health. However, we were surprised to discover that rights and freedoms mediated these effects. Our findings showed that the effects of SES and social capital on physical health were fully mediated through democratic rights and freedoms. However, the mental health model indicated that rights and freedoms completely mediated the effects of SES and only partially mediated the effects of social capital. The models suggest different origins for mental and physical health outcomes. We named the models, the “*rights and freedoms gradient of health*” to acknowledge this latter construct’s crucial role in determining health outcomes. To this end, “*rights and freedoms*” denotes political rights, civil liberties, and freedom from corruption. It is likely that our findings may have come to light because, as far as we know, this is the first study to simultaneously examine the effects of SES, social capital, and rights and freedoms on health outcomes using pathways models.

The mediating role of rights and freedoms has significant implications that deserve further discussion. Most previous studies investigating the effects of rights and freedoms focused on one or two physical health variables – usually life expectancy or infant mortality rates (Dasgupta, 1990; Altman and Castiglioni, 2009). The present study, instead, incorporated three variables of mental health and three of physical health outcomes. Our results are also consistent with previous findings that suggest that SES (Wilkinson and Picket, 2009), social capital (Elgar, 2010), and democratic rights and freedoms (Dasgupta, 1990; Altman and Castiglioni, 2009) are all independently associated with better health outcomes. First, a large body of evidence in support of the “*social inequality argument*” contends that increasing a nation’s SES, in terms of overall wealth (Wickrama and Mulford, 1996) with an equitable distribution of wealth, is associated with better health outcomes (Wilkinson and Picket, 2009). Second, many previous studies support the “*rights and liberties argument*” in that democratic rights and freedoms positively affect physical health, such as decreasing infant mortality rates and increasing life expectancies (Dasgupta, 1990; Franco et al., 2004; Altman and Castiglioni, 2009). However, few studies have examined the effects of rights and freedoms on mental health. One study, though, has shown that when civil liberties increase, suicide rates decrease (Jungeilges and Kirchgässner, 2002). Third, cross-national evidence from the “*civil society and social capital argument*” suggests that social capital is positively related to better health outcomes (Elgar, 2010). Therefore, our findings are consistent with past studies that independently associate SES, rights and freedoms, and social capital with increased health performance. Unique to the present study is that our findings place these latter constructs into relative context to one another.

Over two decades ago, Dasgupta (1990) refuted the argument that poorer countries cannot afford the “luxury” (p.4) of rights and freedoms. Dasgupta further argued that when individuals have greater rights and freedoms, they also perform better regarding health. In this context, rights and freedoms are thought to permit citizens to exert pressure for beneficial social policy (Korpi, 1989). An implication of this study is that its results support the notion that rights and freedoms may be essential social conditions to support not only child survival, but also child and youth development, as rights and freedoms are reflected in population indicators of mental and physical health outcomes over the life-course.

Our findings suggest that to improve population health, countries must strive to integrate efforts addressing all of these three critical factors, concurrently: socioeconomic conditions, civil society and social capital, and rights and freedoms. This concept has been recognized by the United Nations Development Program (UNDP) in developing their Human Development Index, a composite score that measures life expectancy, education and national wealth, “to emphasize that people and their capabilities should be the ultimate criteria for assessing the development of a country, not economic growth alone” [United Nations Development Programme (UNDP), 2011]. Specifically, the democracy theme of the 2002 UNDP report [United Nations Development Programme (UNDP), 2002] recognized the importance of linking democratic rights and freedoms, human development, and health. Yet, the UNDP does not include a measure of these rights and freedoms in their human development index as their 2002 report notes that these concepts “are difficult to measure appropriately” (p. 53).

In addition, the 2002 UNDP report states that civil society development is instrumental in building democracy. Yet, the report does not integrate civil society into their concept of mutually reinforcing capabilities (p. 53) which included links between rights and freedoms, SES, and health-education. A similar sentiment was echoed in the WHO’s framework on the social determinants of health [World Health Organization (WHO), 2010] which noted that “social capital occupy a conspicuous (and contested) place in discussions of SDH (social determinants of health)” (p. 7) because “focus on social capital, depending on interpretation, risks reinforcing depoliticized approaches to public health” (p. 7). Our results suggest that civil society (term used by UNDP), or by extension social capital (term used by WHO), must also be integrated into frameworks and concepts that advance health outcomes. In the context of the UNDP and WHO reports, social capital and civil society are similar concepts. As previously noted, social capital can be defined as the associations or networks that generate the cooperation and trust necessary for the realization of common goals (Putnam, 2000). Civil society can be described as the interactions of citizens “which has a life of its own, which is distinctively different from the state and largely autonomous from it” to promote the collective interest (Shils, 1991). From these perspectives, the terms social capital and civil society can both be construed as reflecting power that originates with citizens and strengthens democratic rights and freedoms.

The relationship between social capital and SES was more difficult to interpret. We chose to denote SES as leading to social capital. This decision was somewhat arbitrary as reversing the relation in enlisting social capital leading to SES in the pathway model is equally statistically equivalent. Future research is warranted in uncovering the path. In any event, our findings suggest that social capital and SES are both necessary for the development of rights and freedoms, and consequently health. In this respect, past research has shown that increasing only SES (in terms of GDP per capita) does not lead to better health outcomes (Altman and Castiglioni, 2009).

Interestingly, our findings also suggest that despite their levels of rights and freedoms, some nations are more resilient than others, in terms of countries falling significantly above or below the regression line. For example, when comparing rights and freedoms versus physical health outcomes, Estonia, Ukraine, and Brazil are significantly below the regression line, while Italy and Greece fall significantly above the regression line. Comparatively for mental health, Argentina falls above the regression line and Hungary falls below. These findings raise a wide range of questions as to what might be at play in those resilient and vulnerable countries. Pertaining to resilience, possibilities might include regional variations that exist within a given country (e.g., differences between the north/south, east/west, rural/urban) or decentralization of health care administration and service provision as may be the case in Italy. Pertaining to vulnerability, ethnic fragmentation (Aghion et al., 2004), authoritarian regimes with an abundance of natural resources in poorer countries (Ross, 2001), and past histories of colonialization (Diamond et al., 1987) can impede democratization. Any such hindrance could plausibly have important health implications, given the results of this study. For example, Ukraine was under colonial rule for centuries (Subtelny, 2009) and suffered from a Soviet-orchestrated genocide in 1932–1933 that claimed the lives of several million Ukrainians (Naimark, 2010). The genocide resulted in a decades-long, decreased life expectancy for survivors and their offspring after the genocidal period (Meslé et al., 2012).

Concerning approaches to improve health performance, our model is also consistent with past research on development aid. Specifically, Kosack (2003) concluded that development aid better effects improvements in health (i.e., life expectancy) when combined with increases in rights and freedoms. Kosack further noted that when rights and freedoms are minimal, as in autocracies, aid is ineffective. These latter aid findings are consistent with our model that suggests rights and freedoms provide the core that successively impacts health. Further, our research also contends that improvements in SES and social capital are foundational prerequisites for the growth of rights and freedoms. By extension, our results suggest that development aid targeting improvements in SES and social capital would also benefit growth in rights and freedom. The latter would, then, improve mental and physical health outcomes.

Our results also have implication concerning current thought on mental health trends. For example, Thachuk (2011) wrote a critical analysis of the recent trend to label mental illness as being exclusively biological in origin. Our findings suggest that mental health (such as suicide, alcohol and tobacco dependencies), to a large extent, also has strong social determinants.

Lastly, our models hold for the variables and countries used in this study. As our sample contained countries located in Europe, North and South America, and the South Caucasus, further study of nations from other regions of the world and other indicators of mental and physical health is necessary. Determining if an additional step, like good governance, exists between rights-freedoms and health outcomes is also warranted. As our findings are cross-sectional, they do not imply causation. However, our results are consistent with studies that have demonstrated causation (Dasgupta and Weale, 1992) in rights and freedoms leading to better health outcomes.

## Conflict of Interest Statement

The authors declare that the research was conducted in the absence of any commercial or financial relationships that could be construed as a potential conflict of interest.
